# Advanced machine learning for innovative drug discovery

**DOI:** 10.1186/s13321-025-01061-w

**Published:** 2025-08-08

**Authors:** Igor V. Tetko, Djork-Arné Clevert

**Affiliations:** 1 Institute of Structural Biology, Molecular Targets and Therapeutics Center, Helmholtz Munich - Deutsches Forschungszentrum Für Gesundheit Und Umwelt (GmbH), 86764 Neuherberg, Germany; 2 BIGCHEM GmbH, Valerystr. 49, 85716 Unterschleißheim, Germany; 3 Machine Learning Research, Pfizer, Friedrichstraße 110, 10117 Berlin, Germany

## Abstract

This editorial presents an analysis of the articles published in the *Journal of Cheminformatics* Special Issue “AI in Drug Discovery”. We review how novel machine learning developments are enhancing structural-based drug discovery; providing better forecasts of molecular properties while also improving various elements of chemical reaction prediction. Methodological developments focused on increasing the accuracy of models via pre-training, estimating the accuracy of predictions, tuning model hyperparameters while avoiding overfitting, in addition to a diverse range of other novel and interesting methodological aspects, including the incorporation of human expert knowledge to analysing the susceptibility of models to adversary attacks, were explored in this Special Issue. In summary, the Special Issue brought together an excellent collection of articles that collectively demonstrate how machine learning methods have become an essential asset in modern drug discovery, with the potential to advance autonomous chemistry labs in the near future.

**Graphical Abstract:**

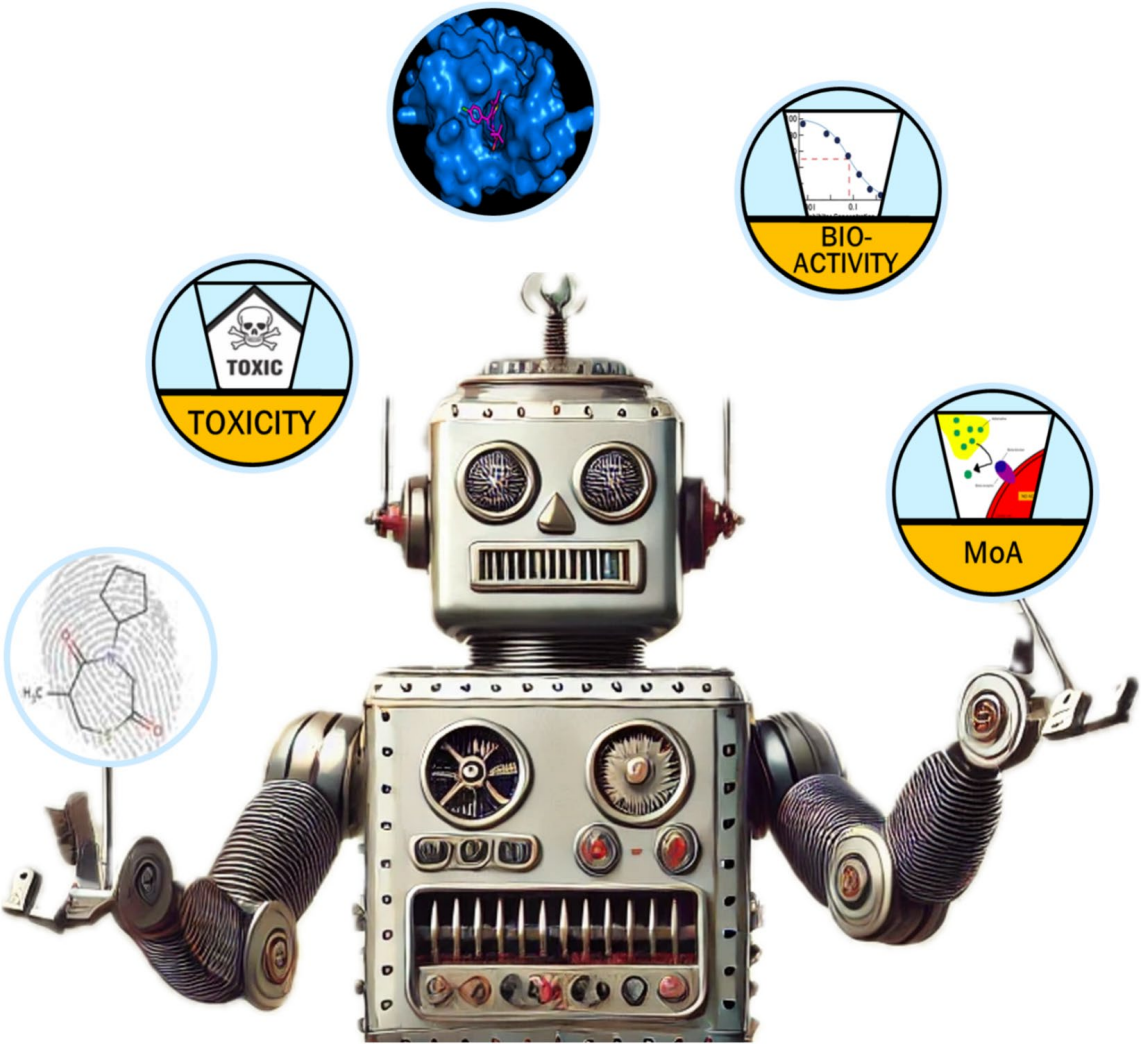

The special issue “AI in Drug Discovery” was organised in connection to a workshop of the same name organised during the 33rd International Conference on Artificial Neural Networks (ICANN2024) conference https://e-nns.org/icann2024. It has received a lot of interest and attention, attracting 63 submissions from 20 countries, of which 26 were published as articles in the journal. Here, we critically review these contributions, which covered various aspects of new developments in machine learning (ML) and chemoinformatics methods in chemistry and drug discovery. We also organised the Tox24 challenge within the framework of ICANN2024. Readers interested in evaluating the recent developments in ML methods to predict toxicity of chemical compounds are invited to read about it in a dedicated article by Eytcheson and Tetko [1].

## Structural-based drug discovery: binding site, docking and scoring functions

A critical step in structure-based drug discovery is the identification of binding pockets, which can be used to develop new active molecules. The (Contrastive Learning and Pre-trained Encoder for Small Molecule Binding) CLAPE-SMB method by Wang et al [2] predicts protein-DNA binding sites using only sequence data. The method demonstrated better or comparable performance in comparison to other methods—even those of them that used 3D information. Interestingly, using focal loss [3] to address data imbalance (since binding sites correspond only to less than 5% of all aminoacids) did not provide a significant improvement.

Once binding sites are identified, users traditionally use docking tools, such as AutoDock [4], to correctly score top poses to identify active molecules. In contrast to traditional docking methods (using force-field based or empirical scoring functions) Gnina [5] uses Convolutional Neural Networks to score such poses. A novel edition of Gnina (v1.3) by McNutt et al [6] retrained the CNN scoring function on an updated docking dataset and introduced knowledge-distilled CNN scoring to increase inference speed. Importantly, a new scoring function for covalent docking was also added, thus extending functionality of the software. Another novel scoring function based on constructing weighted colored subgraphs from the 3D structure of protein–ligand complexes was proposed by Mukta et al. [7]. The authors converted protein ligand complexes to 3D sub-graphs based on SYBYL atom types both for ligands and proteins. Eigenvalues and eigenvectors of sub-graphs were used to generate almost 17 k descriptors (such as sum, mean, max, etc. of eigenvalues). The descriptors were further analysed by gradient boosting trees to develop a regression model—the AGL-EAT-Score (Algebraic Graph Learning with Extended Atom-Type Scoring Function) for predicting binding affinities. Of course, successful application of AGL-EAT-Score and Gnina 1.3 as a whole still depends on the successful identification of true binding poses.

Do current methods correctly identify interactions between small molecules and proteins? The importance of direct assessment of the interactions of molecules with the protein, such as pharmacophores, in addition to physical plausibility of ligand placement was highlighted by Errington et al. [8] The authors concluded that classical methods produced poses that were better at recovering the considered types of interaction and suggested incorporating explicit protein–ligand interaction fingerprints or pharmacophore-sensitive loss to the training of ML models. Such constraints are not directly modelled, e.g. in DeepTGIN by Wang et al., [9] which predicts binding affinity using Transformers [10] and Graph Isomorphism Networks [11]. This multimodal architecture efficiently learns and combines features of ligand (represented as graph), pocket in addition to capturing global characteristics of the protein (both represented as sequence) to achieve its high accuracy. The attention scores do allow visualisation and interpretation of the interactions, which are important for designing novel compounds, but incorporation of additional physical validation of predicted poses could be a promising extension and enhancement of this study.

To our knowledge, the generative model PoLiGenX by Le et al [12] is one of the first to directly address correct pose prediction by conditioning of the ligand generation process on reference molecules located within a specific protein pocket. This strategy allowed the authors to generate ligands with favorable poses that have reduced steric clashes and lower strain energies compared to those generated with other diffusion models. The pharmacophore-sensitive information actually corresponds to human expert knowledge since generation of pharmacophores is usually based on expert analysis of interactions of ligands with protein. Nahal et al [13] further analysed how leveraging a human expert’s knowledge can be used to improve active learning by using their feedback to refine selection of molecules. Human insights allowed better navigation of chemical space and generation of chemicals with more favourable properties.

While interpretations of models using deep learning methods based on SMILES representations, such as Transformers, may not always be consistent [14], group graphs, which are based on a substructure-level molecular representation, developed by Cao [15] allowed unambiguous interpretation of importance of groups for molecular properties predictions. This representation also increased the accuracy of models while decreasing training time.

## Prediction of properties

Of course, for computational studies, like those mentioned above, researchers have to rely on prediction properties of molecules. In this respect, the development of highly accurate Absorption, Distribution, Metabolism, Excretion, and Toxicity (ADMET) models is very important [16]. AttenhERG by Yang et al., [17] which is based on the Attentive FP algorithm [18] has achieved the highest accuracy in a benchmarking study against different external datasets while also allowing interpretation of which atoms contribute most to the toxicity of chemicals. In some cases, hERG toxicity is detected during the later preclinical phase in drug development, when preparing the Investigational New Drug (IND) Application dossier, or even after a drug is approved in the clinic. CardioGenAI by Kyro et al [19] was developed for the early identification of drugs likely to exhibit hERG toxicity and to redesign such drugs to reduce the risk of hERG toxicity while preserving their pharmacological activity. The authors used an autoregressive transformer to generate valid molecules conditioned on the molecular scaffold and physicochemical properties which are further filtered based on models predicting hERG properties. The authors demonstrate their framework by re-engineering several drugs with known hERG liability. Drug-induced liver injury (DILI) is another important toxicological end-point for computational toxicology [20]. DILI is a complex property and can be linked to many different pathways, such as cholestasis, leading to bile acid accumulation in the liver and resulting in hepatotoxicity. StreamChol developed by Rodríguez-Belenguer [21] provides a user-friendly web-based tool to estimate potential toxicity of compounds with respect to this endpoint. Of course, undesired properties of compounds are not necessarily limited to ADMET. E-GuARD developed by Palmacci [22] was designed to predict compounds likely to interfere with biological assays (frequent hitters). The authors mentioned a scarcity of such data, which are also highly imbalanced (e.g., only 0.7–3.3% of compounds are interfering with luciferase [23]. The authors used artificial data augmentation to address data imbalance thus allowing their model to improve its performance by learning not only from experimental data but also from newly generated compounds.

Toxicity can be also a desired property in molecules, i.e. those used for cancer treatments, such as photoactivated chemotherapy. Vigna et al [24] developed a model based on different 2D fingerprints to estimate propensity of transition metal-based complexes to absorb light in the therapeutic window. The use of ML allowed the authors to significantly increase speed of calculations compared to traditional Time-Dependent Density Functional Theory (TDDFT) calculations, while interpretation of models enabled them to make informed modifications to the chemical structure so that they could absorb light. It should also be mentioned that recent advances in machine learning can be directly used instead of traditional DFT [25] calculations by learning accurate and transferable potential for organic molecules, which can be of several magnitude orders faster [26]. The learning of potentials requires data points to be stored for discrete representations of the electron density as grids, for which uniform grids are frequently used. However, the data points can contain very large variations in density values (from 10^–20^ to 10 [4]) which complicate neural network training. To address this issue Ushenin et al [27] propose a core suppression model that reduces the amplitude of core orbitals and allows for better convergence of neural network models. The authors also developed the Lebedev-Angular Grid Network (LAGNet) architecture which stores data points as a standard grid—specifically, a combination of radial and Lebedev (angular) grids—which allowed the authors to decrease storage space requirements and computation costs.

## Analysis and benchmarking machine learning methods

In order for a compound to find use as a drug, it should have certain physico-chemical and ADMET properties. The prediction of these properties is difficult due to the limited amount of experimental data, as well as the frequency of experimental measurements errors and data imbalance. Several studies have investigated whether modern deep learning can contribute better models. There are many data splitting strategies that have been proposed in these studies and using one or another approach may provide different results. Guo et al. [28] found that the Uniform Manifold Approximation and Projection (UMAP) split provided more challenging and realistic benchmarks for model evaluation than other traditional methods, such as Butina splits, scaffold splits and random splits. Graph Neural Networks, such as ChemProp [29, 30] can be used to model physico-chemical and ADMET properties of compounds, and these methods frequently give excellent performances. However, the question of whether learned representations can provide better performances still remains. The authors of ChemProp decided to investigate whether a combination of deep neural networks with a well-developed package of Mordred [31] descriptors, fastprop [32], can provide similar results to this method. The results obtained when testing this algorithm on several sets showed similar performances, but the use of fastprop yielded results considerably faster (around 10x). The authors also suggested that end-users use it with the default hyperparameters, since their extensive optimisation can result in overfitting, in particular for small sets. The latter problem was exemplified in another publication [33] with respect to solubility prediction studies [34]. Tetko et al [33] showed that using a preselected set of hyperparameters could produce models with similar or even better accuracy than those obtained using grid optimisation for ChemProp [30] and Attentive Fingerprint [18]. Importantly, the results obtained using pre-selected hyperparameters were calculated around 10,000 × faster than the results obtained using optimised parameters. Moreover, another method, Transformer CNN [35] yielded a significantly higher performance compared to both of these graph neural methods. Interestingly, the authors of fastprop also compared their method with Transformer CNN but did not use SMILES augmentation (which is an essential feature of the method). While such use resulted in potential underperformance of Transformer CNN, it still had an accuracy similar to both fastprop and ChemProp methods.

The pre-training of models is important for increasing the accuracy of their predictions for downstream tasks. The pre-training can be done in many different ways, e.g., by predicting canonical SMILES based on augmented (sometimes also called random or enumerated [36]) SMILES, which is done with Transformer CNN [35]. Incorporation of calculated properties into pre-training can be beneficial for certain models. Fallani et al [37] analysed the pre-training of a Graphormer [38], which is a Transformer for graphs. The authors found that pretraining of models on quantum-chemical properties contributed better models for ADMET datasets. A similar conclusion on the importance of data pertaining was reached by Masood et al. [39] Their VitroBERT model pre-trained on in vitro data showed a significant improvement for highly imbalanced DILI tasks.

While properties of compounds can be predicted, not all predictions have the same accuracy. An estimation of accuracy of predictions could be done by, e.g., defining a distance to model function, which measures similarity of molecules to the training set/model and calibrating it using it cross-validation or/and test sets [40]. Friesacher et al [41] investigated several different strategies, as well as the influence of model hyper-parameter tuning, which can provide the best model calibration. Interestingly, the authors found that using a novel Bayesian uncertainty estimation method allowed them to obtain models both with higher calibration as well as accuracy. In another study Masood et al [42] used a transformer-based BERT model to reliably estimate uncertainty and improve active learning.

The possibility of sharing information without sharing data has been a topic of careful investigation in drug discovery, in particular when using federated learning [43, 44]. Earlier investigations have also explored the sharing of data using descriptors and concluded that it may indeed be possible to reverse engineer molecules from descriptors [45], which was later confirmed experimentally [46]. An alternative method using surrogate data was proposed [45] and was recently used to share CYP3A4 inhibition data [47]. However, does sharing of models also lead to the data leakage? Krüger et al [48] used recent developments in cryptography to evaluate vulnerabilities across different molecular representations and algorithms. The authors found that using various attacks the adversary can identify molecules from the training set and, in particular, underrepresented compounds, which are usually the most valuable (e.g., active compounds in virtual screening). The use of representation learning graph neural networks significantly reduced vulnerability to these attacks.

## Reaction predictions

Two articles submitted to the Special Issue focused on reaction prediction. In the first study, Torren-Peraire et al [49] developed a benchmark set and a strategy for identifying complex routes with multiple target molecules sharing common intermediates, thus allowing for the synthesis of 30% more compounds simultaneously than is possible using traditional multi-step synthesis planning. The acceleration of inference times to decrease computational costs is an important problem, in particular when considering the effect of deep learning computations on CO2 emissions and climate change [50]. Andronov et al [51] achieved over 3 × faster inference in reaction product prediction tasks with no loss in accuracy when using speculative decoding, which proposes several tokens simultaneously, with the model deciding whether or not it can accept them. Considering the urgent need for data, Vangala et al [52] proposed using LLMs to extract high quality data from patent documents by collecting 26% more new reactions compared to existing tools.

While the articles published in this Special Issue describe various neural network architectures, applications as well as use cases, of course, they do not cover the diversity of developments within this area of research. However, this Special Issue also has a dedicated review of various deep learning chemical language models by Flores-Hernandez and Martinez-Ledesma [53], which provides readers with a systematic analysis of the current developments in the field.

In conclusion, we see very active development of new machine learning approaches for different aspects of drug discovery. These methods are becoming more powerful and successfully competing with various traditional computational chemistry approaches, such as docking, DFT, machine learning methods based on traditional descriptors, etc. The explanation of models’ predictions, as well as incorporation of expert knowledge, are also very important development and progress in these fields is continuing, as was highlighted in a previous editorial [54]. The widespread use of deep learning methods has brought novel issues to the attention of the scientific community, such as the need to decrease computational resources and carbon footprint and to understand security issues when sharing models developed with different molecular representations. The widespread use of computational predictions, in connection with modern hardware implementations to advance autonomous chemistry labs, is likely to be the next big development as machine learning methods continue to progress.

## References

[1] Eytcheson SA, Tetko IV (2025) Which modern AI methods provide accurate predictions of toxicological endpoints? Analysis of Tox24 challenge results. Chem Res Toxicol. 10.1021/acs.chemrestox.5c00273 10.1021/acs.chemrestox.5c00273 40779334

[2] Wang J, Liu Y, Tian B (2024) Protein-small molecule binding site prediction based on a pre-trained protein language model with contrastive learning. J Cheminformatics 16:125 10.1186/s13321-024-00920-2 PMC11542454 39506806

[3] Cui Y, Jia M, Lin T-Y, Song Y, Belongie S (2019) Class-balanced loss based on effective number of samples. IEEE, New York

[4] Trott O, Olson AJ (2010) AutoDock Vina: improving the speed and accuracy of docking with a new scoring function, efficient optimization, and multithreading. J Comput Chem 31:455–461 19499576 10.1002/jcc.21334 PMC3041641

[5] McNutt AT et al (2021) GNINA 1.0: molecular docking with deep learning. J Cheminformatics 13:43 10.1186/s13321-021-00522-2 PMC8191141 34108002

[6] McNutt AT, Li Y, Meli R, Aggarwal R, Koes DR (2025) GNINA 1.3: the next increment in molecular docking with deep learning. J Cheminformatics 17:28 10.1186/s13321-025-00973-x PMC11874439 40025560

[7] Mukta FT, Rana MM, Meyer A, Ellingson S, Nguyen DD (2025) The algebraic extended atom-type graph-based model for precise ligand–receptor binding affinity prediction. J Cheminformatics 17:10 10.1186/s13321-025-00955-z PMC11756177 39844277

[8] Errington D, Schneider C, Bouysset C, Dreyer FA (2025) Assessing interaction recovery of predicted protein-ligand poses. J Cheminformatics 17:76 10.1186/s13321-025-01011-6 PMC12090448 40389970

[9] Wang G et al (2024) DeepTGIN: a novel hybrid multimodal approach using transformers and graph isomorphism networks for protein-ligand binding affinity prediction. J Cheminformatics 16:147 10.1186/s13321-024-00938-6 PMC11684089 39734235

[10] Vaswani A et al (2017) Attention is all you need. ArXiv170603762 Cs. arXiv preprint arXiv:1706.03762, 10.48550/arXiv.1706.03762

[11] Xu K, Jegelka S, Hu W, Leskovec J (2019) How powerful are graph neural networks? arXiv preprint arXiv:181000826. 10.48550/arXiv.1810.00826

[12] Le T, Cremer J, Clevert D-A, Schütt KT (2025) Equivariant diffusion for structure-based de novo ligand generation with latent-conditioning. J Cheminformatics 17:90 10.1186/s13321-025-01028-x PMC12126868 40450349

[13] Nahal Y et al (2024) Human-in-the-loop active learning for goal-oriented molecule generation. J Cheminformatics 16:138 10.1186/s13321-024-00924-y PMC11629536 39654043

[14] Hartog PBR, Krüger F, Genheden S, Tetko IV (2024) Using test-time augmentation to investigate explainable AI: inconsistencies between method, model and human intuition. J Cheminformatics 16:39 10.1186/s13321-024-00824-1 PMC10993590 38576047

[15] Cao P-Y et al (2024) Group graph: a molecular graph representation with enhanced performance, efficiency and interpretability. J Cheminformatics 16:133 10.1186/s13321-024-00933-x PMC11606038 39609909

[16] Tetko IV, Bruneau P, Mewes H-W, Rohrer DC, Poda GI (2006) Can we estimate the accuracy of ADME-Tox predictions? Drug Discov Today 11:700–707 16846797 10.1016/j.drudis.2006.06.013

[17] Yang T et al (2024) AttenhERG: a reliable and interpretable graph neural network framework for predicting hERG channel blockers. J Cheminformatics 16:143 10.1186/s13321-024-00940-y PMC11668031 39716240

[18] Xiong Z et al (2020) Pushing the boundaries of molecular representation for drug discovery with the graph attention mechanism. J Med Chem 63:8749–8760 31408336 10.1021/acs.jmedchem.9b00959

[19] Kyro GW, Martin MT, Watt ED, Batista VS (2025) CardioGenAI: a machine learning-based framework for re-engineering drugs for reduced hERG liability. J Cheminformatics 17:30 10.1186/s13321-025-00976-8 PMC11881490 40045386

[20] de GarciaLomana M, Gadaleta D, Raschke M, Fricke R, Montanari F (2025) Predicting liver-related in vitro endpoints with machine learning to support early detection of drug-induced liver injury. Chem Res Toxicol 38:656–671 40064588 10.1021/acs.chemrestox.4c00453 PMC12015958

[21] Rodríguez-Belenguer P, Soria-Olivas E, Pastor M (2025) StreamChol: a web-based application for predicting cholestasis. J Cheminformatics 17:9 10.1186/s13321-024-00943-9 PMC11752685 39838478

[22] Palmacci V et al (2025) E-GuARD: expert-guided augmentation for the robust detection of compounds interfering with biological assays. J Cheminformatics 17:64 10.1186/s13321-025-01014-3 PMC12042382 40301942

[23] Ghosh D, Koch U, Hadian K, Sattler M, Tetko IV (2018) Luciferase advisor: high-accuracy model to flag false positive hits in luciferase HTS assays. J Chem Inf Model 58:933–942 29667823 10.1021/acs.jcim.7b00574

[24] Vigna V, Cova TFGG, Pais AACC, Sicilia E (2025) Prediction of Pt, Ir, Ru, and Rh complexes light absorption in the therapeutic window for phototherapy using machine learning. J Cheminformatics 17:1 10.1186/s13321-024-00939-5 PMC11702227 39757232

[25] Kohn W (1999) Nobel lecture: electronic structure of matter–-wave functions and density functionals. Rev Mod Phys 71:1253–1266

[26] Smith JS, Isayev O, Roitberg AE (2017) ANI-1: an extensible neural network potential with DFT accuracy at force field computational cost. Chem Sci 8:3192–3203 28507695 10.1039/c6sc05720a PMC5414547

[27] Ushenin K et al (2025) LAGNet: better electron density prediction for LCAO-based data and drug-like substances. J Cheminformatics 17:65 10.1186/s13321-025-01010-7 PMC12042566 40301997

[28] Guo Q, Hernandez-Hernandez S, Ballester PJ (2025) UMAP-based clustering split for rigorous evaluation of AI models for virtual screening on cancer cell lines. J Cheminformatics 17:94 10.1186/s13321-025-01039-8 PMC12153141 40495205

[29] Heid E et al (2024) Chemprop: a machine learning package for chemical property prediction. J Chem Inf Model 64:9–17 38147829 10.1021/acs.jcim.3c01250 PMC10777403

[30] Yang K et al (2019) Analyzing learned molecular representations for property prediction. J Chem Inf Model 59:3370–3388 31361484 10.1021/acs.jcim.9b00237 PMC6727618

[31] Moriwaki H, Tian Y-S, Kawashita N, Takagi T (2018) Mordred: a molecular descriptor calculator. J Cheminformatics 10:4 10.1186/s13321-018-0258-y PMC5801138 29411163

[32] Burns JW, Green WH (2025) Generalizable, fast, and accurate DeepQSPR with fastprop. J Cheminformatics 17:73 10.1186/s13321-025-01013-4 PMC12076996 40361252

[33] Tetko IV, van Deursen R, Godin G (2024) Be aware of overfitting by hyperparameter optimization! J Cheminformatics 16:139 10.1186/s13321-024-00934-w PMC11629497 39654058

[34] Meng J et al (2022) Boosting the predictive performance with aqueous solubility dataset curation. Sci Data 9:71 35241693 10.1038/s41597-022-01154-3 PMC8894363

[35] Karpov P, Godin G, Tetko IV (2020) Transformer-CNN: swiss knife for QSAR modeling and interpretation. J Cheminformatics 12:17 10.1186/s13321-020-00423-w PMC7079452 33431004

[36] Bjerrum EJ (2017) SMILES enumeration as data augmentation for neural network modeling of molecules. arXiv170307076. 10.48550/arXiv.1703.07076

[37] Fallani A et al (2025) Pretraining graph transformers with atom-in-a-molecule quantum properties for improved ADMET modeling. J Cheminformatics 17:25 10.1186/s13321-025-00970-0 PMC11869672 40016793

[38] Ying, C. *et al.* Do transformers really perform bad for graph representation? in proceedings of the 35th international conference on neural information processing systems Article 2212 (Curran Associates Inc., 2021).

[39] Masood A et al (2025) VitroBERT - modeling DILI by pretraining BERT on in vitro data. J Cheminformatics. 10.1186/s13321-025-01048-7 10.1186/s13321-025-01048-7 PMC12326598 40770816

[40] Tetko IV et al (2008) Critical assessment of QSAR models of environmental toxicity against Tetrahymena pyriformis: focusing on applicability domain and overfitting by variable selection. J Chem Inf Model 48:1733–1746 18729318 10.1021/ci800151m

[41] Friesacher HR, Engkvist O, Mervin L, Moreau Y, Arany A (2025) Achieving well-informed decision-making in drug discovery: a comprehensive calibration study using neural network-based structure-activity models. J Cheminformatics 17:29 10.1186/s13321-025-00964-y PMC11881400 40045403

[42] Masood MA, Kaski S, Cui T (2025) Molecular property prediction using pretrained-BERT and Bayesian active learning: a data-efficient approach to drug design. J Cheminformatics 17:58 10.1186/s13321-025-00986-6 PMC12020163 40270038

[43] Heyndrickx W et al (2024) MELLODDY: cross-pharma federated learning at unprecedented scale unlocks benefits in QSAR without compromising proprietary information. J Chem Inf Model 64:2331–2344 37642660 10.1021/acs.jcim.3c00799 PMC11005050

[44] Bassani D, Brigo A, Andrews-Morger A (2023) Federated learning in computational toxicology: an industrial perspective on the effiris hackathon. Chem Res Toxicol 36:1503–1517 37584277 10.1021/acs.chemrestox.3c00137 PMC10523574

[45] Tetko IV, Abagyan R, Oprea TI (2005) Surrogate data–a secure way to share corporate data. J Comput Aided Mol Des 19:749–764 16267691 10.1007/s10822-005-9013-3

[46] Le T, Winter R, Noé F, Clevert D-A (2020) Neuraldecipher–reverse-engineering extended-connectivity fingerprints (ECFPs) to their molecular structures. Chem Sci 11:10378–10389 34094299 10.1039/d0sc03115a PMC8162443

[47] Fluetsch A, Trunzer M, Gerebtzoff G, Rodríguez-Pérez R (2024) Deep learning models compared to experimental variability for the prediction of CYP3A4 time-dependent inhibition. Chem Res Toxicol 37:549–560 38501689 10.1021/acs.chemrestox.3c00305

[48] Krüger FP, Östman J, Mervin L, Tetko IV, Engkvist O (2025) Publishing neural networks in drug discovery might compromise training data privacy. J Cheminformatics 17:38 10.1186/s13321-025-00982-w PMC11948693 40140934

[49] Torren-Peraire P et al (2025) Improving route development using convergent retrosynthesis planning. J Cheminformatics 17:26 10.1186/s13321-025-00953-1 PMC11869726 40016850

[50] Hartog PBR, Westerlund AM, Tetko IV, Genheden S (2025) Investigations into the efficiency of computer-aided synthesis planning. J Chem Inf Model 65:1771–1781 39889203 10.1021/acs.jcim.4c01821 PMC11863376

[51] Andronov M, Andronova N, Wand M, Schmidhuber J, Clevert D-A (2025) Accelerating the inference of string generation-based chemical reaction models for industrial applications. J Cheminformatics 17:31 10.1186/s13321-025-00974-w PMC11895308 40065398

[52] Vangala SR et al (2024) Suitability of large language models for extraction of high-quality chemical reaction dataset from patent literature. J Cheminformatics 16:131 10.1186/s13321-024-00928-8 PMC11590295 39593165

[53] Flores-Hernandez H, Martinez-Ledesma E (2024) A systematic review of deep learning chemical language models in recent era. J Cheminformatics 16:129 10.1186/s13321-024-00916-y PMC11571686 39558376

[54] Tetko IV, Engkvist O (2020) From big data to artificial intelligence: chemoinformatics meets new challenges. J Cheminformatics 12:74 10.1186/s13321-020-00475-y PMC7747384 33339533

